# Serum tumor marker and CT body composition scoring system predicts outcomes in colorectal cancer surgical patients

**DOI:** 10.1007/s00330-024-10849-7

**Published:** 2024-06-24

**Authors:** Mingming Song, Zhihao Liu, Feihong Wu, Tong Nie, Yixin Heng, Jiaxin Xu, Ning Huang, Xiaoyu Wu, Yinghao Cao, Gang Hu

**Affiliations:** 1 Department of General Surgery, The Second People’s Hospital of Hefei Affiliated to Bengbu Medical University, Hefei, 230011 China; 2 Department of General Surgery, The Second People’s Hospital of Hefei, Hefei, 230011 China; 3 China Medical University, Shenyang, 110122 China; 4 Department of Radiology, Union Hospital, Tongji Medical College, Huazhong University of Science and Technology, Wuhan, 430022 China; 5 Hubei Province Key Laboratory of Molecular Imaging, Wuhan, 430022 China; 6 Department of General Surgery, The First Affiliated Hospital of Shihezi University, Shihezi, 832000 P.R. China; 7 Department of Gastrointestinal Surgery, Union Hospital, Tongji Medical College, Huazhong University of Science and Technology, Wuhan, 430022 China; 8 Cancer Center, Union Hospital, Tongji Medical College, Huazhong University of Science and Technology, Wuhan, 430022 China; 9 National Local Joint Laboratory for Advanced Textile Processing and Clean Production, Wuhan Textile University, Wuhan, 430073 China

**Keywords:** Colorectal cancer, Body composition, Computed tomography, Overall survival, Disease-free survival

## Abstract

**Objective:**

To investigate the prognostic value of preoperative body composition and serum tumor markers (STM) in patients undergoing surgical treatment for colorectal cancer (CRC) and to establish the prognostic score for patients with CRC.

**Methods:**

This study enrolled 365 patients (training set 245, validation set 120) with CRC who underwent surgical resection. The predictive value of various body composition features and STM for determining CRC prognosis were compared. A novel index score based on the independent risk factors from Cox regression for CRC patients was established and evaluated for its usefulness.

**Results:**

Multivariate Cox regression showed that low skeletal muscle radiodensity (SMD) (*p* = 0.020), low subcutaneous fat area (SFA) (*p* = 0.029), high carcinoembryonic antigen (CEA) (*p* = 0.008), and high alpha-fetoprotein (AFP) (*p* = 0.039) were all independent prognostic factors for poor overall survival (OS). The multifactorial analysis indicated that high intermuscular fat area (IMFA) (*p* = 0.033) and high CEA (*p* = 0.009) were independent prognostic factors for poor disease-free survival (DFS). Based on these findings, two scoring systems for OS and DFS were established in the training datasets. CRC patients who scored higher on the new scoring systems had lower OS and DFS (both *p* < 0.001) as shown in the Kaplan–Meier survival curves in the training and validation datasets.

**Conclusion:**

In predicting the prognosis of CRC patients, SFA and SMD are superior to other body composition measurements. A scoring system based on body composition and STM can have prognostic value and clinical applicability.

**Clinical relevance statement:**

This scoring system, combining body composition and serum tumor markers, may help predict postoperative survival of CRC patients and help clinicians make well-informed decisions regarding the treatment of patients.

**Key Points:**

- *Colorectal cancer prognosis can be related to body composition*.
- *High intermuscular fat area and CEA were independent prognostic factors for poor disease-free survival*.
- *This scoring system, based on body composition and tumor markers, can prognosticate for colorectal cancer patients*.

## Introduction

Colorectal cancer (CRC) is a prevalent malignant tumor globally, and incidence has been increasing in recent years, posing a significant threat to human health [1, 2]. Despite recent improvements in multidisciplinary treatments such as surgery, chemotherapy, and radiotherapy, the mortality rate for patients with CRC remains high–especially for those with distant metastases or postoperative recurrence [3]. Therefore, early adoption of accurate prognostic factors, followed by effective and targeted interventions to improve prognosis, is essential. The tumor lymph node metastasis (TNM) classification and staging system is widely used in clinical practice to assess the prognosis of patients with CRC by evaluating the depth of the tumor, the extent of lymph node metastasis, and distant metastasis [4]. However, the prognosis of patients with CRC is not determined by TNM staging alone.

Previous studies have indicated that muscle and fat tissue measured by computed tomography (CT) images can indicate the prognosis of CRC patients treated with surgical resection [5–8]. In the past, weight loss and body mass index (BMI) were frequently used as indicators of nutritional decline and poor prognosis [9, 10], however, BMI alone is not an adequate indicator of the difference between fat and muscle mass, or between visceral adipose tissue and skeletal muscle [11], as these have opposing prognostic effects [12, 13]. CT imaging, which has been demonstrated to accurately reflect different types of adipose tissue and muscle mass [14], is routinely used in the clinical management of patients with CRC. Research has indicated that low SMA and high visceral-to-total fat ratios are linked to worse colon cancer outcomes [15]. Conversely, high SMI at the L3 and umbilical levels have been significantly correlated with improved OS and DFS (All *p* < 0.05) [16, 17]. In addition, serum tumor markers (STM) are widely used to assess the prognosis of CRC. For example, elevated pre-operative CEA levels are associated with worse disease-free survival (DFS) and overall survival (OS), as well as an increased risk of recurrence and metastasis [18, 19]. In recent years, with the deepening understanding of tumor mechanisms and the development of molecular biology technology, the detection of tumor markers has become a common method for monitoring tumor recurrence and metastasis, as well as for assessing prognosis and survival [20].

There is an increasing interest in using body composition or STM to achieve personalized predictions in patients with CRC. We postulate that combinations of multiple markers may have greater predictive value than a single body composition feature or STM. There are almost no relevant studies on metrics that combine CT-quantified body composition and STM. Nie et al found that high SMD and serum tumor markers (CA72-4) were significantly associated with better OS, and a model constructed on the basis of these two and other metrics had an AUC of up to 0.848 [21]. However, the authors only investigated the predictive value of body composition and serum tumor markers in patients with rectal cancer and did not give any specific use of the model in the clinical setting. Therefore, we propose a novel prognostic score based on these two factors to address this issue and explore its value in predicting prognosis of patients with CRC.

## Material and method

### Patients

**Fig. 1:**
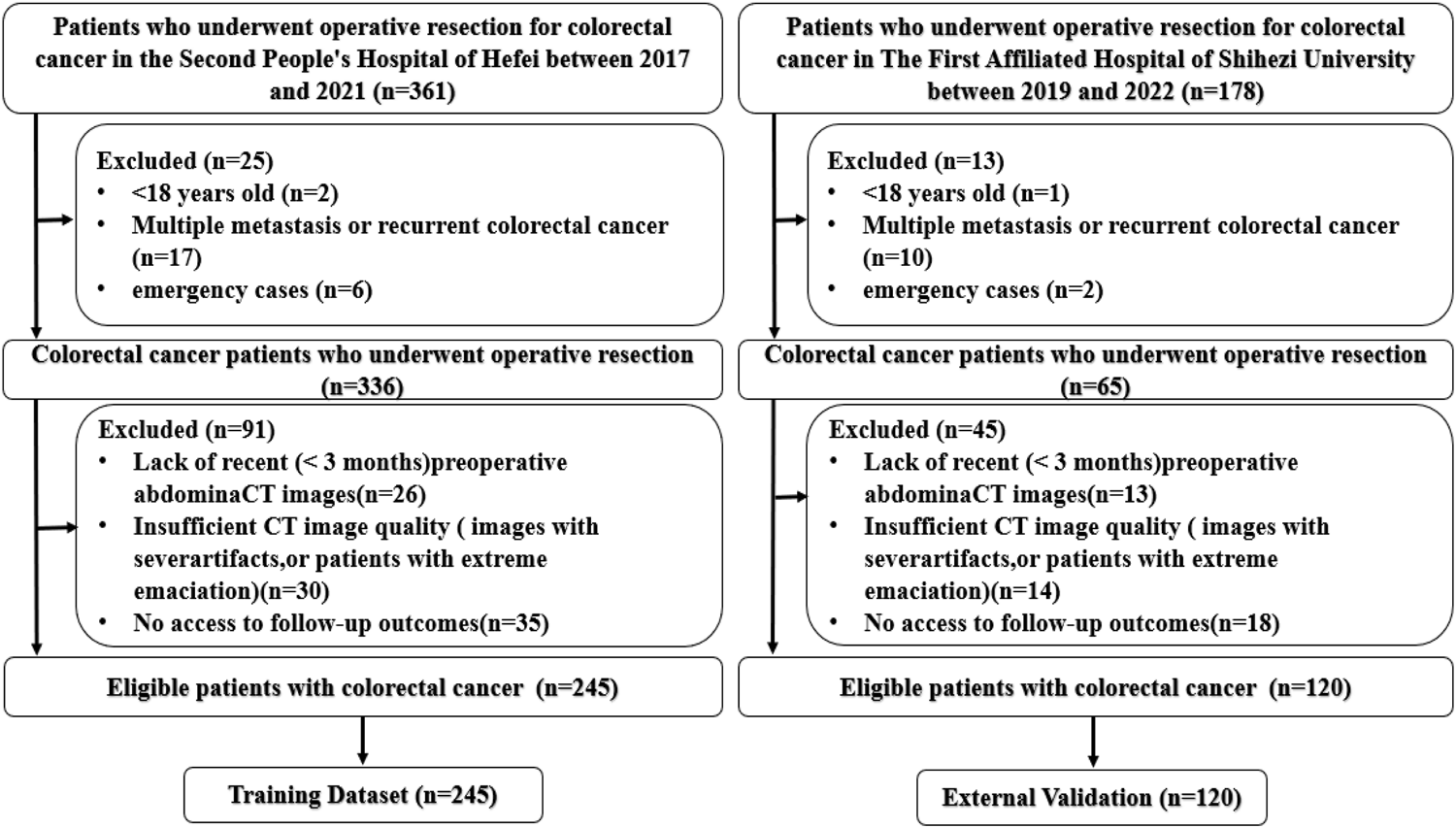
Flowchart of the population

This was a retrospective cohort study of CRC patients who underwent surgical resection with curative intent at the Second People’s Hospital of Hefei between January 2017 and May 2021 was used as a training dataset. It conducted an external validation dataset by screening the CRC patients with the same criteria from The First Affiliated Hospital of Shihezi University between April 2019 and March 2022. Inclusion criteria were listed: (1) age of 18 years or older and pathologic confirmation of CRC; (2) surgical resection with curative intent; (3) abdominal CT scan within 3 months before surgery. Exclusion criteria were composed of the following: (1) emergency cases; (2) multiple colorectal metastases or recurrence; (3) loss of follow-up or inability to obtain follow-up outcomes; (4) incomplete clinical data; (5) no neoadjuvant therapy (neoadjuvant therapy will change the body composition parameters). Ultimately, a total of 245 patients were eligible for the training dataset and 120 patients for the external validation dataset (Fig. 1).

The study was approved by the Ethics Committee of the Second People’s Hospital of Hefei City (No. 2023-S127), and all patient-related medical data were kept confidential. The study was conducted according to the tenets of the Declaration of Helsinki. A waiver of informed consent has been granted by the Ethics Committee.

### Data collection and classification of variables

Clinical information and perioperative data were collected for each patient, including gender, age, BMI, tumor location, tumor size, TNM stage (T stage, N stage, M stage), carcinoembryonic antigen (CEA), alpha-fetoprotein (AFP) and glycoantigens (CA19-9, CA125, CA-724). Follow-up was conducted until July, 2023, through telephone contact or consultation with patients to determine their status: whether they survived or not, and whether they survived with or without recurrence. OS was defined as the time from surgery to death or the follow-up cut-off date, and DFS was defined as the time from the day of surgery to tumor recurrence, metastasis, or the follow-up cut-off date.

### Body composition assessment

**Fig. 2:**
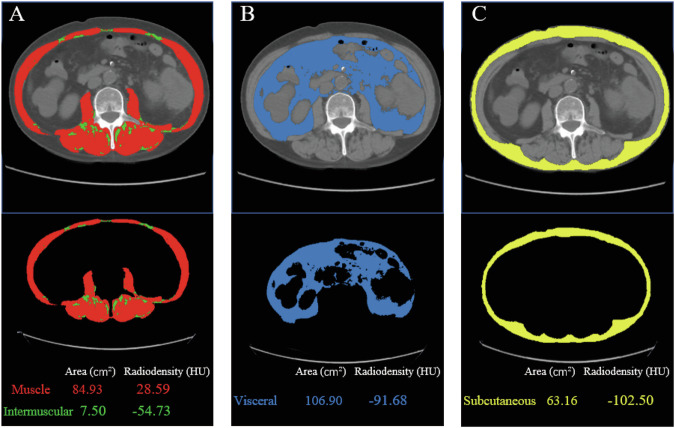
Body morphometric evaluations of abdominal fat and muscle areas at the L3 level. Axial slices at the L3 level of a male. **A** Skeletal muscle area (in red) is 84.93 cm^2^; Intermuscular fat area (in green) is 7.50 cm^2^; **B** Visceral fat area (in blue) is 106.90 cm^2^; **C** Subcutaneous fat area (in yellow) is 63.16 cm^2^

Abdominal CT images of patients with CRC were obtained from their electronic medical records within three months prior to surgery. A single image at the level of the third lumbar vertebra (L3) was selected for quantifying muscle mass (Fig. 2A) and adipose tissue (Fig. 2B, C), as the cross-sectional area of skeletal muscle and adipose tissue at this level strongly correlates with tissue volume at the whole-body level [22, 23]. All relevant images were analyzed using Tomovision’s SliceOmatic (v5.0, Magog, Quebec, Canada). The CT HU thresholds were −29 to +150 for skeletal muscle area (SMA), −150 to −50 for visceral fat area (VFA), and −190 to −30 for subcutaneous fat area (SFA) and intermuscular fat area (IMFA) [24]. The software automatically generated skeletal muscle density (SMD) as the average CT value of the muscle region within the area of interest. Skeletal muscle mass index (SMI) was calculated by dividing the total muscle cross-sectional area by the square of the body height. As gender differences affect the distribution of body composition, we measured the optimal cut-off values for each body composition index separately for males and females using receiver operating characteristic (ROC) curve analysis, and then grouped patients accordingly.

### Statistical analysis

Count data is presented as percentages *N* (%) and measurements are presented as mean ± standard deviation. Baseline data were compared between groups using the chi-square test or Fisher’s exact test with qualitative variables. The Kaplan–Meier survival curves were plotted, and the differences were analyzed using the log-rank test. Univariate and multivariate analyses were performed using a Cox proportional hazards model to identify the independent prognostic factors. Receiver operating characteristic (ROC) curve analysis was used to identify the optimal cut-off values of body composition and STM. A scoring system was developed by assigning one point to each of the poor independent prognostic factors identified in the multifactorial Cox proportional hazards model, and then summing the scores (methodology was determined following data collection). ROC curve analysis was used to compare the predictive power of the new scoring system with that of the individual metrics and TNM stage. We used IBM-SPSS software, version 26.0 (IBM Corp.) and R software (version 4.3.0) for data analysis. All tests were 2-sided, and a *p* value < 0.05 was considered statistically significant.

## Results

### Patients’ characteristics

The study included a total of 245 patients as a training set, comprising 141 males (57.6%) and 104 females (42.4%). Furthermore, the external validation set consisted of a total of 120 patients. Table 1 provides detailed information on the general clinical characteristics of the patients in the training set. A higher proportion of patients in the surviving group had tumors located in the colon compared to the non-surviving group (43.4% vs 60.9%, *p* = 0.011). Patients with TNM staging in stages I and II, N staging in stage N0, and M staging in stage M0 had a higher survival rate (all *p* < 0.05). In terms of body composition and STM, patients with high VFA (24.9% vs 11.8%, *p* = 0.020), high SFA (44.4% vs 28.9%, *p* = 0.022), and high SMD (42.0% vs 25.0%, *p* = 0. 011), as well as the levels of CEA < 4.39 (68.6% vs 44.7%, *p* < 0.001), the levels of CA19-9 < 18.86 (74.0% vs 44.7%, *p* < 0.001), the levels of AFP *p* < 6.56 (88.8% vs 69.7%, *p* < 0.001), and the levels of CA125 < 15.07 (71.6% vs 56.6%, *p* = 0.021) had a higher survival rate. There was no significant difference between the training set and the validation set (Table S1).

**Table 1:** Patient’s baseline characteristics by survivor status in training dataset

Characteristic		Survivors (*n* = 169)	Non-survivors (*n* = 76)	*p*-value
Sex				0.836
	Male	98 (58.0%)	43 (56.6%)	
	Female	71 (42.0%)	33 (43.4%)	
Age (years)				0.274
	< 65	61 (36.1%)	22 (28.9%)	
	≥ 65	108 (63.9%)	54 (71.1%)	
BMI (kg/m^2^)				0.151
	< 18.5	19 (11.2%)	6 (7.9%)	
	18.5–25	121 (71.6%)	49 (64.5%)	
	≥ 25	29 (17.2%)	21 (27.6%)	
Tumor location				**0.011**
	Rectal	66 (39.1%)	43 (56.6%)	
	Colon	103 (60.9%)	33 (43.4%)	
Tumor size (cm)				0.682
	≤ 2.5	15 (8.9%)	8 (10.5%)	
	≥ 2.5	154 (91.1%)	68 (89.5%)	
Stage				**<** **0.001**
	I	34 (20.1%)	4 (5.3%)	
	II	80 (47.3%)	24 (31.6%)	
	III	48 (28.4%)	30 (39.5%)	
	IV	7 (4.1%)	18 (23.7%)	
T Stage				0.052
	T1	3 (1.8%)	1 (1.3%)	
	T2	34 (20.1%)	5 (6.6%)	
	T3	66 (39.1%)	38 (50.0%)	
	T4	66 (39.1%)	32 (42.1%)	
N Stage				**<** **0.001**
	N0	117 (69.2%)	31 (40.8%)	
	N1	48 (28.4%)	38 (50.0%)	
	N2	4 (2.4%)	7 (9.2%)	
M Stage				**<** **0.001**
	M0	162 (95.9%)	58 (76.3%)	
	M1	7 (4.1%)	18 (23.7%)	
VFA				**0.020**
	Low	127 (75.1%)	67 (88.2%)	
	High	42 (24.9%)	9 (11.8%)	
IMFA				0.926
	Low	110 (65.1%)	49 (64.5%)	
	High	59 (34.9%)	27 (35.5%)	
SMA				0.169
	Low	60 (35.5%)	34 (44.7%)	
	High	109 (64.5%)	42 (55.3%)	
SFA				**0.022**
	Low	94 (55.6%)	54 (71.1%)	
	High	75 (44.4%)	22 (28.9%)	
SMD				**0.011**
	Low	98 (58.0%)	57 (75.0%)	
	High	71 (42.0%)	19 (25.0%)	
SMI				0.837
	Low	91 (53.8%)	42 (55.3%)	
	High	78 (46.2%)	34 (44.7%)	
CEA (μg/L)				**<** **0.001**
	< 4.39	116 (68.6%)	34 (44.7%)	
	≥ 4.39	53 (31.4%)	42 (55.3%)	
CA19-9 (U/mL)				**<** **0.001**
	< 18.86	125 (74.0%)	34 (44.7%)	
	≥ 18.86	44 (26.0%)	42 (55.3%)	
AFP (μg/L)				**<** **0.001**
	< 6.56	150 (88.8%)	53 (69.7%)	
	≥ 6.56	19 (11.2%)	23 (30.3%)	
CA125 (U/mL)				**0.021**
	< 15.07	121 (71.6%)	43 (56.6%)	
	≥ 15.07	48 (28.4%)	33 (43.4%)	

Bold was used to highlight values that were statistically significant (*P* < 0.05)

*CEA* carcino-embryonic antigen, *AFP* alpha-fetoprotein, *CA19-9* CA125, carbohydrate antigen, *VFA* visceral fat area, *IMFA* intermuscular fat area, *SMA* skeletal muscle area, *SFA* subcutaneous fat area, *SMD* skeletal muscle density, *SMI* skeletal muscle mass index

### Factors associated with long-term survival

The univariate analysis of OS revealed that CEA, CA19-9, AFP, CA125, VFA, SFA, and SMD were associated with the prognosis of patients with CRC patients in the training set. The multivariate analysis indicated that higher SFA (Male ≥ 102.33 cm^2^, Female ≥ 118.90 cm^2^) (HR = 0.555, 95% CI 0.328–0.940, *p* = 0.029) and higher SMD (Male ≥ 36.09 HU, Female ≥ 42.29 HU) (HR = 0.527, 95% CI 0.308–0.902, *p* = 0.020) were significantly associated with better OS. While high CEA level ( ≥ 4.39 μg/L) (HR = 1.909, 95% CI 1.181–3.084, *p* = 0.008) and high AFP level ( ≥ 6.56 μg/L) (HR = 1.770, 95% CI 1.029–3.044, *p* = 0.039) were identified as independent prognostic factors for poor overall survival in CRC patients in the training set (Table 2). The COX univariate analysis of DFS demonstrated that CEA, CA19-9, AFP, CA125, IMFA and SMA were associated with the prognosis of CRC patients. In the meantime, multivariate analysis identified that high IMFA (Male ≥ 14.39 cm^2^, Female ≥ 27.78 cm^2^) (HR = 1.769, 95% CI 1.049–2.983, *p* = 0.033) and high CEA level (HR = 2.207, 95% CI 1.217–4.000, *p* = 0.009) were independent prognostic factors for poor DFS in CRC patients in the training set (Table 3).

**Table 2:** Univariate and multivariate analysis of factors associated with overall survival in training dataset

Variables		Univariate analysis		Multivariate analysis	
		HR (95% CI)	*p* value	HR (95% CI)	*p* value
Sex					
	Male	1			
	Female	0.949 (0.603–1.495)	0.823		
Age (years)					
	< 65	1			
	≥ 65	1.349 (0.821–2.217)	0.238		
CEA (μg/L)					
	< 4.39	1		1	
	≥ 4.39	2.411 (1.532–3.796)	**<** **0.001**	1.909 (1.181–3.084)	**0.008**
CA19-9 (U/mL)					
	< 18.86	1		1	
	≥ 18.86	2.586 (1.640–4.076)	**<** **0.001**	1.589 (0.951–2.653)	0.077
AFP (μg/L)					
	< 6.56	1		1	
	≥ 6.56	2.284 (1.399–3.730)	**0.001**	1.770 (1.029–3.044)	**0.039**
CA125 (U/mL)					
	< 15.07	1		1	
	≥ 15.07	1.901 (1.206–2.995)	**0.006**	1.230 (0.759–1.996)	0.401
VFA					
	Low	1		1	
	High	0.478 (0.238–0.959)	**0.038**	0.804 (0.382–1.690)	0.564
IMFA					
	Low	1			
	High	1.139 (0.711–1.825)	0.588		
SMA					
	Low	1			
	High	0.669 (0.425–1.053)	0.082		
SFA					
	Low	1		1	
	High	0.533 (0.324–0.875)	**0.013**	0.555 (0.328–0.940)	**0.029**
SMD					
	Low	1		1	
	High	0.482 (0.286–0.813)	**0.006**	0.527 (0.308–0.902)	**0.020**
SMI					
	Low	1			
	High	1.003 (0.638–1.577)	0.990		

Bold was used to highlight values that were statistically significant (*p* *<* 0.05)

*CEA* carcino-embryonic antigen, *AFP* alpha-fetoprotein, *CA19-9* CA125 carbohydrate antigen, *VFA* visceral fat area, *IMFA* intermuscular fat area, *SMA* skeletal muscle area, *SFA* subcutaneous fat area, *SMD* skeletal muscle density, *SMI* skeletal muscle mass index

**Table 3:** Univariate and multivariate analysis of factors associated with disease-free survival in training dataset

Variables		Univariate analysis		Multivariate analysis	
		HR (95% CI)	*p* value	HR (95% CI)	*p* value
Sex					
	Male	1			
	Female	0.941 (0.564–1.570)	0.815		
Age (years)					
	< 65	1			
	≥ 65	1.068 (0.624–1.829)	0.810		
CEA (μg/L)					
	< 4.39	1		1	
	≥ 4.39	2.725 (1.531–4.850)	**0.001**	2.207 (1.217–4.000)	**0.009**
CA19-9 (U/mL)					
	< 18.86	1		1	
	≥ 18.86	2.19 (1.288–3.724)	**0.004**	1.474 (0.835–2.603)	0.181
AFP (μg/L)					
	< 6.56	1		1	
	≥ 6.56	1.806 (1.004–3.248)	**0.048**	1.461 (0.763–2.626)	0.270
CA125 (U/mL)					
	< 15.07	1		1	
	≥ 15.07	1.915 (1.126–3.259)	**0.017**	1.568 (0.893–2.753)	0.117
VFA					
	Low	1			
	High	1.059 (0.638–1.759)	0.824		
IMFA					
	Low	1		1	
	High	1.960 (1.180–3.255)	**0.009**	1.769 (1.049–2.983)	**0.033**
SMA					
	Low	1		1	
	High	0.531 (0.307–0.916)	**0.023**	0.609 (0.350–1.060)	0.080
SFA					
	Low	1			
	High	0.970 (0.574–1.639)	0.908		
SMD					
	Low	1			
	High	0.801 (0.481–1.335)	0.394		
SMI					
	Low	1			
	High	0.881 (0.518–1.498)	0.639		

*CEA* carcino-embryonic antigen, *AFP* alpha-fetoprotein, *CA19-9* CA125 carbohydrateantigen, *VFA* visceral fat area, *IMFA* intermuscular fat area, *SMA* skeletal muscle area, *SFA* subcutaneousfat area, *SMD* skeletal muscle density, *SMI* skeletal muscle mass index

Bold was used to highlight values that were statistically significant (*p* < 0.05)

**Fig. 3:**
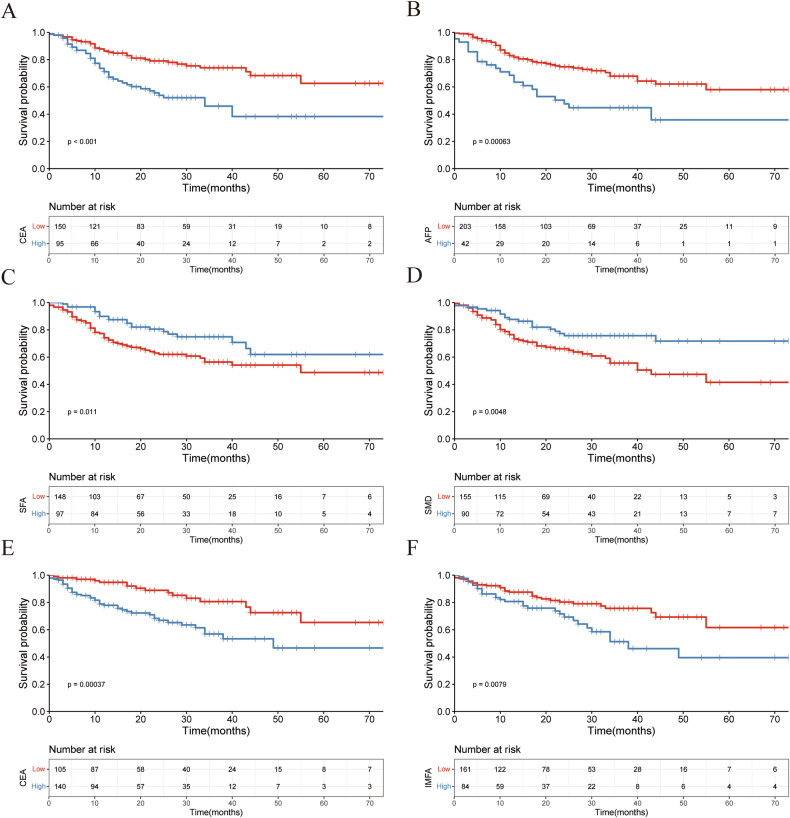
Kaplan-Meier survival curves for overall survival and disease free survival at the L3 level in training dataset. The Kaplan-Meier survival curves for overall survival of patients grouped by low and high CEA (**A**), AFP (**B**), SFA (**C**), SMD (**D**), and disease free survival of patients grouped by low and high CEA (**E**), IMFA (**F**) at the L3 level

**Fig. 4:**
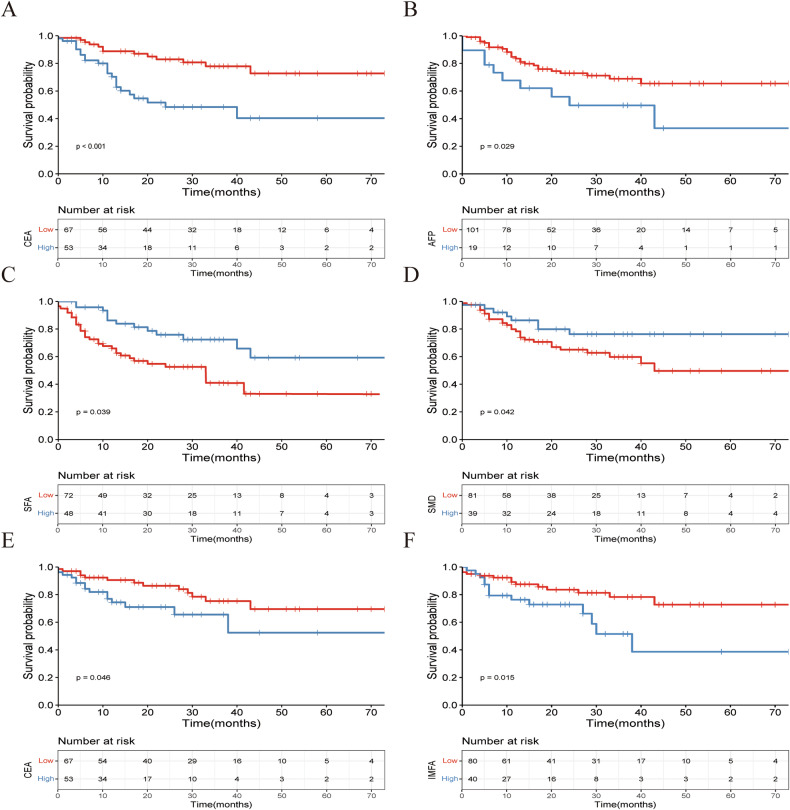
Kaplan-Meier survival curves for overall survival and disease free survival at the L3 level in external validation dataset. The Kaplan-Meier survival curves for overall survival of patients grouped by low and high CEA (**A**), AFP (**B**), SFA (**C**), SMD (**D**), and disease free survival of patients grouped by low and high CEA (**E**), IMFA (**F**) at the L3 level

The Kaplan-Meier survival curves indicate that CRC patients with lower levels of CEA and AFP in the training (Fig. 3A, B) and validation (Fig. 4A, B) sets had better OS rates. Additionally, patients from the training (Fig. 3C, D) and validation (Fig. 4C, D) set in the high SFA and SMD group also showed better overall survival (all *p* < 0.05). Furthermore, lower levels of CEA and IMFA were significantly associated with better DFS (training set: Fig. 3E, F; validation set: Fig. 4E, F) (all *p* < 0.05).

### Creation of the novel index

Considering that OS represents the overall survival and DFS represents the progression of the disease, we have constructed two separate scoring systems. According to the results of the multivariate analysis, among the factors included in the regression (including age, sex, body composition and serum tumor markers), lower SFA and lower SMD were significantly associated with poorer OS. High CEA and high AFP were also prognostic factors for poor OS. We focused on these 4 parameters and created a novel index score that was used to predict the OS (Table 4). On multivariate analysis, higher IMFA and higher CEA were significantly associated with poor DFS. We focused on these two parameters and created another novel index score to predict the DFS (Table 4). In the multifactorial analysis, the HR values for each factor were equivalent; therefore, the weights of the factor scores were set to the same value. New index scores were calculated for each patient, ranging from 0–4 (OS) and 0–2 (DFS).

**Table 4:** 4.1 Calculation of the novel index score associated with overall survival (OS). 4.2 Calculation of the novel index score associated with disease-free survival (DFS)

Factor			Score
4.1 (OS)			
CEA (μg/L)	< 4.39		0
	≥ 4.39		1
AFP (μg/L)	< 6.56		0
	≥ 6.56		1
SFA (cm^2^)	Male	Female	
	< 102.33	< 118.9	1
	≥ 102.33	≥ 118.9	0
SMD (HU)	Male	Female	
	< 36.09	< 42.29	1
	≥ 36.09	≥ 42.29	0
Novel index score	Sum of each score of the four factors		0–4
**4.2 (DFS)**			
CEA (μg/L)	< 4.39		0
	≥ 4.39		1
IMFA (cm^2^)	Male	Female	
	< 14.39	< 27.78	0
	≥ 14.39	≥ 27.78	1
Novel index score	Sum of each score of the two factors		0–2

*CEA* carcino-embryonic antigen, *AFP* alpha-fetoprotein, *CA19-9* CA12-5 carbohydrate antigen, *VFA* visceral fat area, *IMFA* intermuscular fat area, *SMA* skeletal muscle area, *SFA* subcutaneous fat area, *SMD* skeletal muscle density, *SMI* skeletal muscle mass index

### Postoperative survival based on the novel index score

**Fig. 5:**
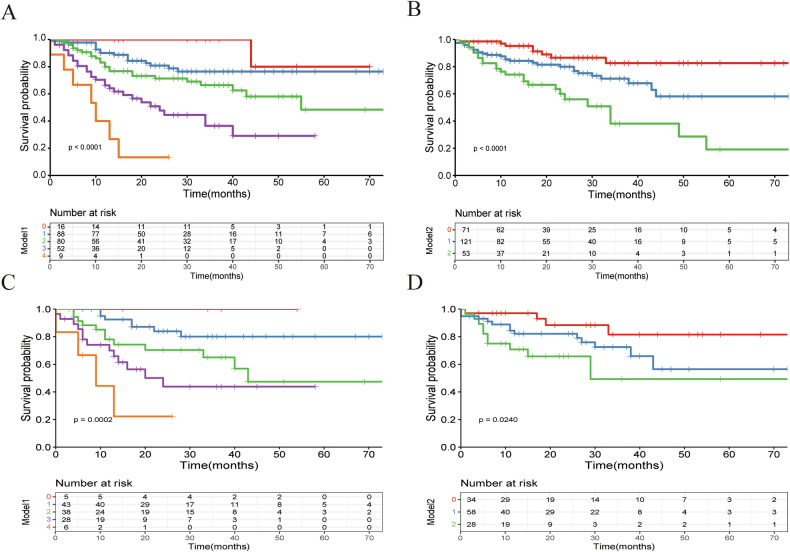
Kaplan-Meier survival curves for novel index. Kaplan–Meier survival curves for overall survival according to each novel index score based on carcinoembryonic antigen (CEA) level, alpha-fetoprotein (AFP), skeletal muscle area (SFA) and subcutaneous fat area (SMD) in training dataset (**A**) and external validation dataset (**C**). Novel index:,0; ,1; ,2; ,3; ,4. Kaplan–Meier survival curves for disease free survival according to each novel index score based on carcinoembryonic antigen (CEA) level, and intermuscular fat area (IMFA) in training dataset (**B**) and external validation dataset (**D**). Novel index: ,0; ,1; ,2

CRC patients with a higher novel index score in the training and validation sets had poorer OS rates (*p* < 0.001) (training set: Fig. 5A; validation set: Fig. 5C) and DFS rates (*p* < 0.001) (training set: Fig. 5B; validation set: Fig. 5D) as shown in the Kaplan–Meier survival curves.

**Fig. 6:**
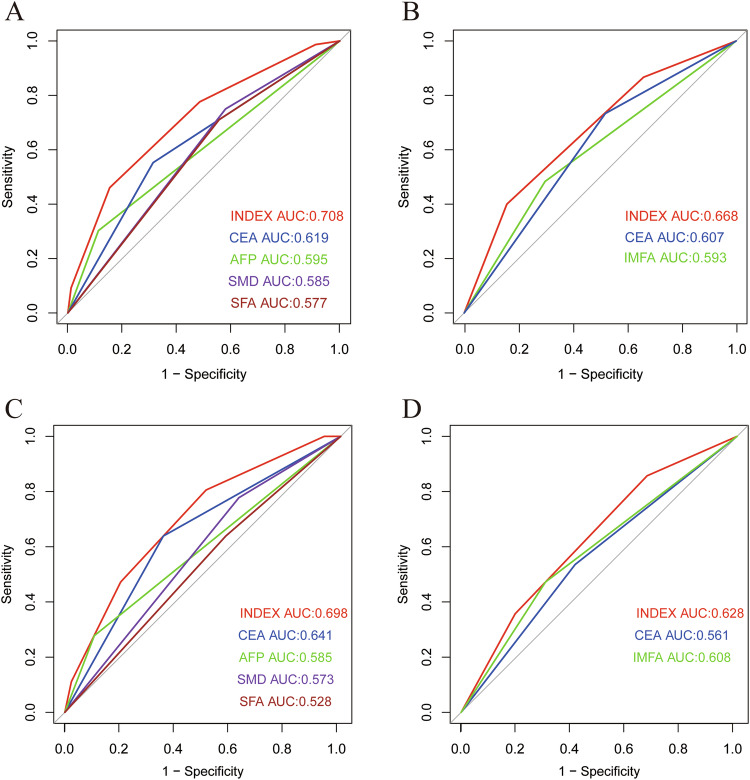
Areas under the receiver operating characteristic curves for overall survival and disease-free survival. Index: CEA, AFP, SFA, SMD at the third lumbar level. The area under the curve of the five indexes for overall survival in the training dataset (**A**) and external validation dataset (**C**). Index: CEA, IMFA at the third lumbar level. The area under the curve of the three indexes for disease-free survival in the training dataset (**B**) and external validation dataset (**D**)

We compared the predictive ability of the new scoring system with a single prognostic biomarker for OS and DFS in CRC patients in the training and validation sets using ROC curve analysis. In the OS group of training set and validation sets, the area under the curve (AUC) of the new index score was 0.708 and 0.698, whereas the AUC of the single prognostic biomarker CEA, AFP, SFA, and SMD of training set were 0.619, 0.595, 0.585, and 0.577 respectively (Fig. 6A), when it comes to the validation set, they were 0.641, 0.585, 0.573, and 0.528, respectively (Fig. 6C). In the DFS group of training set and validation sets, the AUC of the new index score was 0.668 and 0.628, while the AUC of the single prognostic biomarker CEA, IMFA was 0.607, 0.593 (Fig. 6B) and 0.561, 0.608 (Fig. 6D) (all *p* < 0.001). We also compared the predictability of the novel index score with TNM stage for OS and DFS. For OS, the area under the curve value of the novel index score was 0.708, while that of TNM stage was 0.653 (*p* < 0.001) (Fig. S1A). For DFS, the area under the curve value of the novel index score was 0.668, while that of TNM stage was 0.596 (*p* < 0.001) (Fig. S1B). We found that the new scoring system combining body composition and STM had significantly better predictive ability for both OS and DFS than the single prognostic biomarker and TNM stage.

## Discussion

In this study, we established a new scoring system based on body composition measures and STM, analyzed and compared its predictive efficacy with single indicators in both the training set and the external validation set. This is of significant importance for evaluating the prognosis of CRC patients receiving surgical treatment and selecting further adjuvant therapy strategies. The new scoring system shows promising potential in predicting postoperative survival status of CRC patients.

Previous studies have investigated the relationship between body composition and prognosis in cancer patients. However, traditional indicators such as BMI, waist-to-hip ratio, and Eastern Cooperative Performance Statu (ECOG-PS) do not provide detailed quantitative data for clinical reference [25]. Quantitative body composition based on CT can address this issue by easily and accurately calculating and distinguishing various body compositions, including skeletal muscle and different visceral fat tissues, using CT images and software. However, there is still controversy regarding which factors may influence mortality rates. Ebadi et al found that adipose tissue is an independent prognostic factor for mortality in patients with gastrointestinal tumors, respiratory system tumors, and metastatic renal cell carcinoma [26]. Our study on CRC patients indicates that adipose tissue is also an independent prognostic factor, and patients with high SFA and VFA exhibit better overall survival. Simultaneously, Oikawa et al demonstrated in their study on metastatic CRC patients that high subcutaneous adipose tissue is associated with a lower risk of mortality (*p* < 0.025) [27]. Our research confirms a better prognosis in patients with high SFA compared to those with low SFA. Additionally, we observed a significant increase in DFS in patients with high IMFA. We hypothesize that the association between high IMFA and improved prognosis may be related to low muscle mass, as it can indirectly reflect the presence of sarcopenia. However, further research is needed to confirm this hypothesis.

The skeletal muscle component in body composition has also been proven to be closely associated with poor prognosis in cancer patients [28–30]. Lee et al, through their study on CRC patients undergoing curative resection, found that patients with low SMD had lower OS [17]. This observation aligns with our research findings, where we discovered that patients with low SMD had worse prognosis and poorer long-term survival. In cancer patients, excessive protein degradation and/or reduced protein synthesis in skeletal muscle can lead to muscle atrophy and infiltration of intramuscular and intermuscular fat. This is attributed to factors such as inflammation, altered protein metabolism, decreased cell apoptosis, and impaired tissue regeneration [31]. Our results confirm that quantitative measurement of skeletal muscle density and intermuscular fat tissue using CT can serve as an effective method for identifying this adverse prognosis.

Multiple studies have shown that elevated levels of CEA are associated with adverse prognosis in CRC [32, 33]. Combining CEA levels with TNM staging may provide a more accurate prediction of CRC prognosis [34]. While AFP is commonly used in patients with hepatocellular carcinoma [35, 36], there is a lack of literature reporting its utility in CRC patients. In this study, the Cox multivariable survival analysis revealed that elevated levels of AFP and CEA were independent adverse prognostic factors for OS in CRC patients. Therefore, we combined these markers with meaningful body composition variables through a novel scoring system, which demonstrated a high predictive efficacy.

We developed a prognostic model for CRC patients using a scoring system. The cutoff values for the variables included in the scoring system have been previously described in the manuscript (Table 4). Clinicians can efficiently convert continuous variables into categorical variables based on this scoring system, enabling the assessment of prognosis by obtaining the final score for CRC patients. Previous studies on the prognosis of CRC patients have often utilized nomograms [37, 38]. However, this method is not only complex to implement but also requires an explanation of how to interpret the curves, which limits its clinical applicability. In our study, we integrated body composition and STM to establish a novel scoring system. This approach not only demonstrated high predictive efficacy but also proved to be user-friendly and easily interpretable. These advantages facilitate the widespread clinical application of this scoring system.

This study has several limitations. Firstly, it is a retrospective study with a limited number of included patients, which may lead to type II errors and selection bias. Furthermore, our study quantified body composition at the L3 anatomical landmark based on preoperative CT scans in CRC patients. Therefore, these findings may not be applicable to studies that quantify body composition at other anatomical landmarks or use alternative imaging modalities (such as MRI). Nevertheless, our study also possesses numerous strengths. It is the first investigation to combine body composition and STM for predicting postoperative survival in CRC patients, demonstrating high predictive performance in both the training set and the external validation set. Furthermore, it is worth noting that our cohort is based on real-world electronic health record data, so our results are demonstrably generalisable. Meanwhile, future studies will include more prospective work to demonstrate the score’s benefits in daily practice. The parameters required for the scoring system are derived from routine preoperative examinations of patients, offering a cost-effective and easily accessible approach. Therefore, it serves as an important prognostic indicator that can guide treatment decisions, stratify prognosis, and enable early interventions.

In summary, we developed a postoperative survival scoring system for CRC patients by integrating clinically accessible STM and quantified body composition from CT scans. The scoring system exhibited excellent predictive accuracy for the prognosis of CRC patients, providing clinical practitioners with guidance for more precise and targeted clinical decision-making, individualized treatment strategies, and disease management.

## Supplementary information

Electronic Supplementary Material

## Abbreviations

AFP: Alpha-fetoprotein
AUC: Area under the curve
BMI: Body mass index
CEA: Carcinoembryonic antigen
CRC: Colorectal cancer
CT: Computed tomography
DFS: Disease-free survival
IMFA: Intermuscular fat area
OS: Overall survival
ROC: Receiver operating characteristic
SFA: Subcutaneous fat area
SMD: Skeletal muscle radiodensity
SMI: Skeletal muscle mass index
STM: Serum tumor markers
TNM: Tumor-lymph node-metastasis
VFA: Visceral fat area
